# Sweat and odor in sportswear – A review

**DOI:** 10.1016/j.isci.2023.107067

**Published:** 2023-06-08

**Authors:** Yuping Chang, Xungai Wang

**Affiliations:** 1 JC STEM Lab of Sustainable Fibers and Textiles, School of Fashion and Textiles, The Hong Kong Polytechnic University, Hung Hom, Kowloon, Hong Kong; 2 Research Institute for Sports Science and Technology, The Hong Kong Polytechnic University, Hung Hom, Kowloon, Hong Kong

**Keywords:** Chemical property, Materials application, Materials synthesis

## Abstract

Sportswear worn next to the skin is easily soaked by sweat and may become a breeding ground for the microbiome, thus a source of malodor. Malodor can cause social embarrassment and discomfort to both wearer and others. Given the risks current deodorant products pose to nature and human life, the development of sustainable textiles for odor control comes to the forefront. This review introduces the odor-generating mechanism in clothing from the perspectives of perspiration composition and cutaneous microbiome. With the knowledge of the significant role of sweat in odor formation, the sweat distribution of the human body, measurement techniques, and advanced technologies developed for quick-dry function are presented in the second part. Lastly, odor management in sportswear is evaluated, covering the odor-assessing techniques, the effects of various textile materials, and emerging solutions in terms of antibacterial treatment, adsorbent materials, and photocatalytic degradations of odorous compounds. Overall, it is of both personal and social value to develop novel textile materials with odor-control functions by making use of natural materials and fabric designs.

## Graphical abstract

**Figure:**
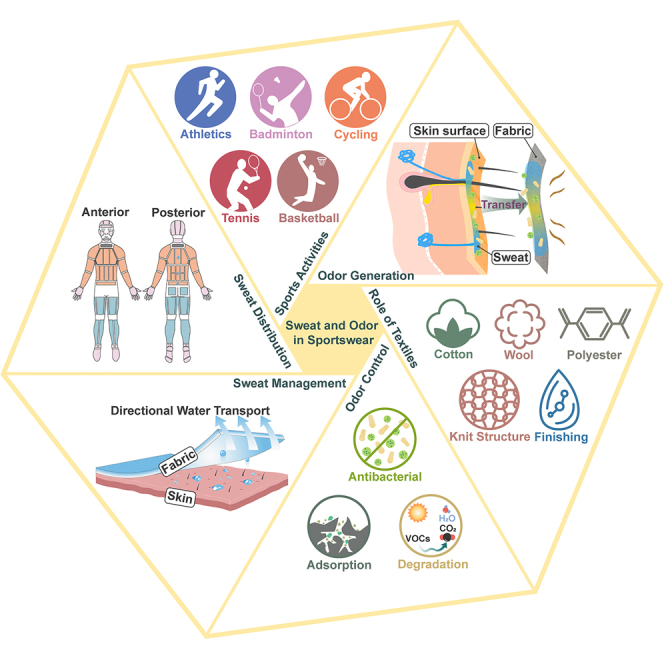

## Introduction

Despite sweating being a natural and essential process of the human body, malodor arising from excessive sweating may cause a tremendous amount of social embarrassment. During physical exercises, the human body needs to consume its stored energy and thus would generate significant heat.^1^ To maintain a safe body temperature, the sweat glands are activated to function as a thermoregulator. Perspiration evaporation can remove excess heat from the skin surface and prevent the human body from hyperthermia.^2^ Axillary odor is usually considered the most pungent body odor, which is closely associated with the profuse apocrine secretions in the armpit. The main strategies of deodorants are antiperspirant, antibacterial and fragrance. Aluminum salts and other metallic salts, such as zirconium, are known for their ability to reduce sweating by physically obstructing glandular ducts, thus are widely used in antiperspirants up to date. The possible link between the use of aluminum antiperspirants with breast cancer has been proposed but remains controversial.^3^^,^^4^ Triclosan, one of the most widely used antibacterial agents, is a broad-spectrum antimicrobial against both Gram-positive and Gram-negative bacteria. For now, it is considered safe for topical use in humans, but it is increasingly detected in the environment, arousing concerns of its harmful impacts on aquatic organisms and its bioaccumulation in food because of its presence in irrigating water.^5^^,^^6^ Triclosan with 18 other antibacterial chemicals, has been banned by the United States Food and Drug Administration (FDA) for use in over-the-counter consumer antiseptic wash products since 2016. The use of deodorant and antiperspirant products has also been found to influence bacterial species richness and composition in the axilla communities, but more need to be done to reveal its consequent impact on the human body.^7^^,^^8^ Masking an unpleasant odor with fragrance might be a simple and quick solution for many. But a light fragrance might find it hard to cover the malodor, whereas an intense fragrance spray can only provide a temporary masking effect, and may cause discomfort or allergies in others. Owing to the rising concerns about the hazardous impact of deodorants on the ecosystem and the risks to human health, researchers are looking for more sustainable alternative solutions.

Clothing has long been found significant in the formation of axillary odor.^9^ Sportswear, which is often exposed to substantial sweat, is particularly prone to generate malodor. The word “sportswear” is defined as “clothes that are worn for sports or other physical activities” in Cambridge Dictionary. But it is far more than that nowadays. With the ever-increasing worldwide interest in active indoor and outdoor activities, sportswear has become a general term covering both clothing and footwear that people wear for physical exercise, leisure, or, even more, for fashion. The passion for sports activities in the modern era poses an increasingly higher requirement for the development of sportswear, such as in sweat management and odor control. To avoid vagueness and misunderstanding, the sportswear we are discussing from the perspective of odor management in this review is narrowed down to the clothing and footwear that people wear next to their skin and are directly exposed to sweat.

This review is aimed at providing a systematic and comprehensive understanding of the odor generation mechanism in clothing because of sweating, and the current and potential strategies targeting sweat management and odor control in sportswear. First and foremost, how odor is generated in sportswear is elaborated from the aspects of perspiration composition and cutaneous microbiome. Given the significant role of sweat in odor formation, the sweating pattern of the human body and the advanced techniques that have been developed for sweat management in sportswear are introduced in the second part. Next, the odor evaluation techniques, the effects of various textile materials, as well as the tackling strategies from the antimicrobial, adsorbent, and photocatalytic degradation perspectives are discussed. In this context, suggestions for the development of sustainable odor-control sportswear in the future are given in the outlook. It is hoped that the need for body odor management could be met without jeopardizing other living creatures or human safety in the next-generation sportswear.

## Generation of odor in sportswear

### Perspiration composition

The human body’s perspiration is a complex mixture of secretions from cutaneous glands, which mainly comprise sweat glands and sebaceous glands. Depending on whether or not the ductal opening is connected to a hair follicle, the sweat glands are roughly classified into two types: eccrine and apocrine.^10^ The eccrine glands open directly to the surface of the skin and account for the highest volume of sweat secretion. As the major type, they are distributed almost across the entire surface of the human body except for lips and glans penis and start to function at the early age of life.^11^ The eccrine sweat is originally a clear, dilute, and odorless fluid, mainly containing water and sodium chloride. Other minor components include micronutrients (e.g., potassium, calcium, magnesium, iron, vitamins), and metabolites (e.g., urea, lactate, amino acids, bicarbonate), with varying concentrations.^12^ It also contains proteins such as immunoglobulins in its minority, taking up less than 1% by weight. The apocrine glands, on the other hand, are associated with hair follicles and therefore are restricted to hairy body areas.^2^ They are mostly distributed in the axilla but also scattered in the mammary, perineal, and genital areas.^2^ The ratio between apocrine and eccrine glands is around 1:1 in the axilla, while being approximately 1:10 in other regions. They are present in the human skin at birth but are not activated until puberty.^2^ The secretion from apocrine glands is a viscous, sterile, and odorless fluid, containing proteins, lipids, steroids, carbohydrates, ammonia, ferric ions, fatty acid, etc.^13^ It should be noted that the apocrine excretions are usually in a mixture with sebum because most of the sebaceous glands are connected with hair follicles as well. Distinguished from the former two, a mixed type named apoeccrine glands was proposed in 1987, which is concentrated in the hairy area of the axillar and has an eccrine-like ductal opening.^14^ It was reported that their excretion composition resembles that of eccrine sweat, therefore will not be elucidated here.^2^^,^^14^

As widely distributed as the sweat glands, sebaceous glands cover most of the human body except for the palms and soles, particularly concentrated in the scalp, forehead, and face areas.^15^ Likewise, they can be categorized into two groups: pilosebaceous glands and free sebaceous glands. The former type is connected with hair follicles whereas the latter is not.^15^ Emphasis will be put on the secretions from the former type here because they account for the majority of the sebaceous glands and their secretions are inevitably in the mixture of perspiration. The discharge of the pilosebaceous glands is sebum, in which triglycerides, diglycerides, and free fatty acids take up ∼57% in weight, followed by wax esters (26%), squalene (12%) and, the least, cholesterol.^15^^,^^16^ The sebaceous gland function in the human body can be regulated by endocrine hormones (such as androgens and estrogens), retinoids, and many other endocrine and nonendocrine compounds.^15^^,^^16^^,^^17^ Despite the complexity of perspiration constituents, apocrine sweat is generally regarded as the primary cause of body odor, providing nutrients for bacterial growth and precursors for odorous compound formation. Eccrine sweat, on the other hand, disperses the apocrine sweat over a larger area and creates a moist environment suitable for the growth of bacteria.

### Cutaneous microbiome

Freshly produced sterile sweat is odorless but the bacterial degradation of precursors in the sweat can make it odorous. There exist trillions of bacteria, fungi, viruses, archaea, and small arthropods on the skin surface forming the skin microbiome.^18^ The microorganisms can be grouped into resident and transient types. The resident microbes are those that are routinely found on the skin, reestablish themselves after perturbation, and are often considered commensal, whereas the transient microbes arise from the environment and only persist for hours to days.^18^ The type and abundance of bacteria are largely dependent on the location and the microenvironment of the skin. The axilla is the region where both eccrine and apocrine glands thrive with sebaceous glands in the mixture, and, therefore, acts as one of the human body areas with the highest density of cutaneous microorganisms.^19^^,^^20^ The axillary microbiome is deemed important in the generation of body odor. The majorities of the bacterium genera in the axilla are Gram-positive Staphylococcus, Corynebacterium, and Cutibacterium,^19^^,^^21^^,^^22^ with the first two dominating the axillary malodor. The Staphylococci are associated with a faint acid, non-apocrine odor, whereas Corynebacteria are more responsible for a pungent, apocrine odor, or bromhidrosis.^7^

The malodor arising from sweat perspiration is mainly based on steroids, long-chain fatty acids, volatile fatty acids, and thioalcohols.^23^ Odorous 16-androstenes are generated from odorless precursor steroids in sweat because of the collective function of different bacteria species, with Corynebacterium being the key type.^23^ Likewise, lipids in perspiration can be catalyzed by secreted lipases into free fatty acids and part of the long-chain fatty acids would be further degraded to volatile fatty acids under the action of Corynebacteria, resulting in malodor.^23^ As one of the major sources of malodor, short-chain and medium-chain volatile fatty acids can be produced in different ways, such as from glycerol and lactic acid by cutibacteria and staphylococci, or from branched aliphatic amino acid by staphylococci, or from epidermis proteins by bacterial proteases.^23^^,^^24^ Thioalcohol, 3-methyl-3-sulfanhexan-1-ol (3M3SH) in particular, is the most pungent volatile component in the axillary malodor. It is generated from the precursor Cys-Gly-3M3SH that is excreted from apocrine glands.^19^^,^^23^

**Figure 1:**
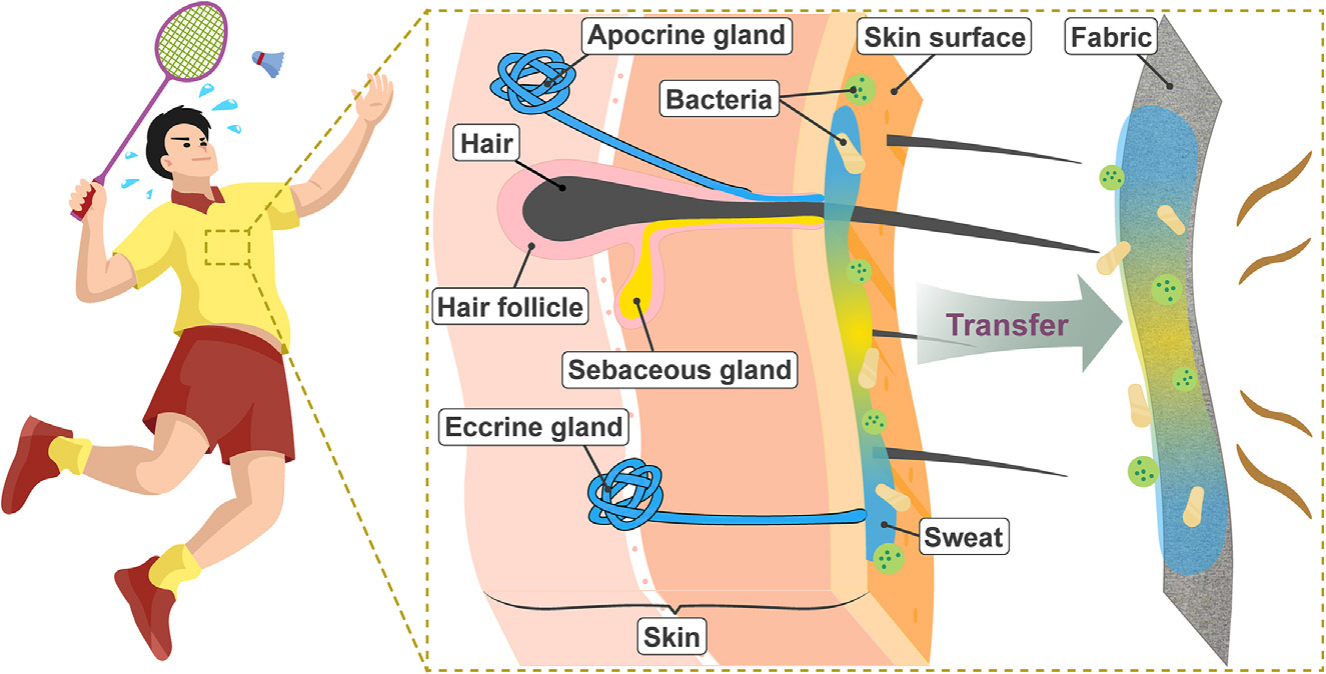
Odor formation in sportswear

In brief, the interplay of skin gland secretions and cutaneous microbiomes in the human body leads to sweat-induced body odor, as shown in Figure 1. The eccrine and sebaceous secretions can promote a nurturing and flourishing environment for the cutaneous microorganisms, and the apocrine secretions can provide precursors for the generation of volatile odorous molecules, whereas the cutaneous microorganisms play the role of the transformer. All of the sweat compositions and microorganisms are easily transferred to the adjacent clothing during physical exercise. The significant role of clothing in axillary odor generation has been recognized in 1953.^9^ It was concluded that even if the axillar is odorless, some odor can develop from the adjacent clothing, especially when apocrine sweating is excessive. Therefore, malodor from sportswear soaked with profuse sweat could be as intense as that from the human body, if not more.

## Sweat analysis in sportswear

### Measurement approach

In light of the significant role of sweat in the odor generation of sportswear, it is of necessity to have a full picture of the sweat loss quantification and the sweat mapping on the human body, especially under exercise-induced load. Diverse methods have been reported to study the sweating pattern of the human body in terms of whole-body sweat rate (WSR), regional sweat rate (RSR), and sweat composition.

For the determination of WSR and electrolyte content from the entire body, a whole-body rinse technique was preferably used.^25^^,^^26^^,^^27^ In brief, subjects and equipment involved should be rinsed thoroughly with deionized water before and after the trial, with the post-rinse water collected for electrolyte analysis via ion chromatography. To diminish the effect of water evaporation from the skin surface, the trials were usually conducted in an isolated chamber consisting of an enclosed plastic bag or capsule, which may in turn lead to high skin hydration, sweat gland ducts blockage, and thus a progressive decrease in sweating.^25^^,^^27^^,^^28^ The whole-body sweat loss can be calculated from the change in nude body mass, corrected for fluid intake, respiratory water loss, and weight loss because of substrate oxidation.^27^^,^^29^ By dividing sweat loss by the body surface area and the duration of the trial, the WSR (in mg·cm^−2^·min^−1^) can be computed. This approach is quite direct and is commonly used in the laboratory, yet it is impractical for the quantification of whole-body sweating in real-life physical activities.^30^ The differences within and between individuals in the respiratory water loss, gas exchange, and metabolic water production make the simple model of sweat loss calculation more or less deviate from the real value.^31^ Novel wearable devices have been developed for sweat loss measurement, which can be classified into hygrometer-based, absorbent-material-based, and microfluidics-based types.^32^ But the wearables can only measure sweat loss in limited body areas, the data of which are usually generalized to whole body regions.

The most commonly used routes to measure RSR are via ventilated capsules and technical absorbents. Typically, the ventilated capsule method is to attach the capsules to the selected sites of the human body, either glued or secured with an elastic strip, and to ventilate them with air at a steady speed.^33^^,^^34^ The relative humidity (RH) and the temperature of the inlet air should be regulated by passing above a saturated lithium chloride solution. With the exhaust air humidity and temperature measured using capacitance hygrometers, the RSR can be derived from changes in RH and temperature of the air passing through the capsule.^34^ The surface area of each ventilated capsule is usually 2–10 cm^2^, and for some particular sites, such as the toe, the medial and lateral aspects of the foot, an exceptionally smaller size of 1.4 cm^2^ was used.^34^^,^^35^^,^^36^^,^^37^^,^^38^ With such small coverage areas on the human body, the generalization of the capsule data to the entire body sweating pattern remained questionable.^36^^,^^39^ By contrast, the technical absorbent method can be applied to a larger region of the human body and was first used by Hevenith et al. in 2008.^36^ It requires the application of absorbent pads, which have a considerable absorption capacity. The size of the pads can be customized based on the anthropometric data of the target body regions. Each absorbent patch should be weighed before and after the test. The RSR (in mg·cm^−2^·min^−1^) can be calculated from the mass change, the surface area, and the application time of the patch. For most areas of the human body, custom-sized plastic sheeting was attached to each pad to prevent the evaporation of sweat, and stretchy long-sleeve T-shirts and trousers were put on to ensure that each pad is in intimate contact with the skin. For the breast area of females, pads with plastic sheeting were attached inside a sports bra.^40^ When foot and hand regions were included, 100% cotton socks and gloves were worn with plastic stretchy socks and latex gloves worn on top.^40^

Comparing the two approaches, each has its own merits and limitations. The ventilated capsules can provide a continuous trace of local sweating. However, their coverage area is limited and is impractical to use for athletes engaging in contact or outdoor sports.^27^ The technical absorbent method enables simultaneous RSR measurement over larger body areas. But the occlusive patches may prevent airflow across the skin surface and create a microenvironment that impacts the sweat rate. A large area of dressing may lead to the accumulation of heat and an increase in body temperature. Also, prolonged application on the skin may induce hidromeiosis. The common solution is to keep the sample time of each absorbent pad under 5 min. In addition, the absorbents require a period before being applied to the human body, hence they can only provide limited data in a specific time zone per experiment.^27^^,^^36^^,^^41^ In general, the technical absorbent approach has become increasingly popular, especially when studying the dynamic sweat distribution of the human body.^39^^,^^42^^,^^43^

All of the sweating pattern measuring techniques aforementioned were performed by trained personnel in laboratory environments, utilizing costly equipment and complex sampling procedures. In the new era, revolutionary skin-based wearable sensors have been used to assess dynamic sweating in a continuous and portable way.^44^^,^^45^^,^^46^ The skin-interfaced wearable sensors for sweat analysis are promising in remote monitoring of health, such as the dynamic metabolic activity of the human body and athletic performance during sports activities. However, this type of sweat monitoring technique, at the primary stage of its development, is deficient from the perspective of rapid collection, sampling, and storage of sweat, high sensitivity of biosensing, energy management, and memory capacity for continuous operation, especially under remote and various environmental conditions.^46^ With its usually portable and compact features, it is more suitable for sweat analysis on limited body surface areas than portraying the sweat distribution across the whole body.

### Body sweat distribution

**Figure 2:**
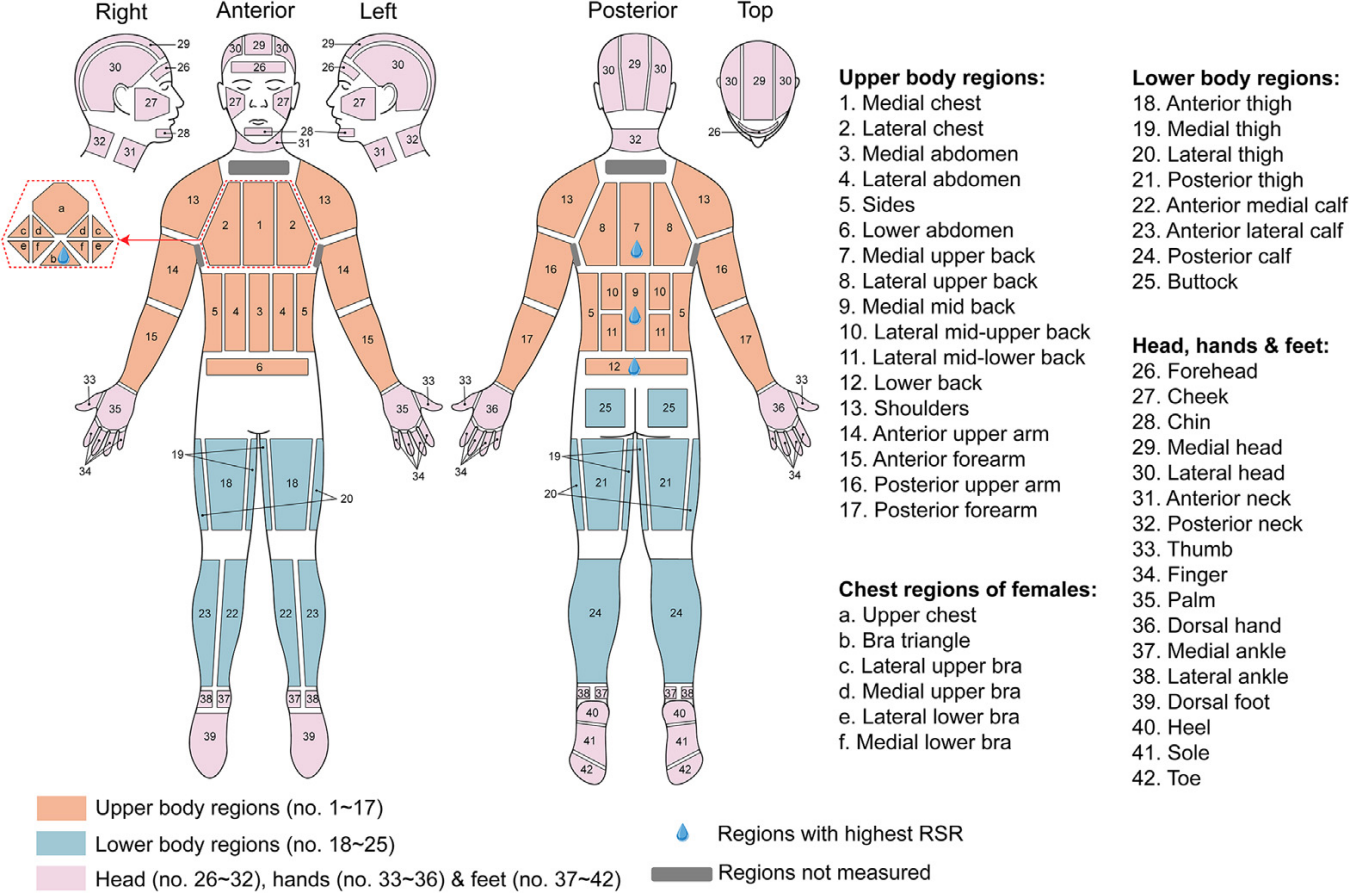
Human body partition (based on whole-body sweat mapping in^40^^,^^41^^,^^43^)

Because odor in sportswear is closely related to sweat, how sweat distributes across the human body should be comprehensively understood. The whole-body mapping of sweat distribution has been intensively studied using the absorbent technique by a research team from Environmental Ergonomics Research Center, Loughborough University, in the UK.^40^^,^^41^^,^^43^ The human body can be divided into upper body regions (UBR), lower body regions (LBR), and head, hand and feet regions (HHFR), for the application of absorbent pads and the analysis of sweat distribution, as illustrated in Figure 2. The whole-body sweat distribution of young Caucasian male athletes was first studied by subjecting participants to running on the treadmill at different exercise intensities.^41^ The exercise intensity was characterized by maximal oxygen consumption (VO_2 max_), with the lower intensity equaling 55% of VO_2 max_ and the higher 75% of VO_2 max_. The medial and lower back (7, 9, 12), and the forehead (26) showed the highest RSR among all regions at both intensities, whereas the lowest RSR mainly lay in the hand regions (33, 34, 35, 36), the plantar foot areas (40, 41, 42), the face regions (27, 28), as well as the upper arm (14, 16) and medial thigh (19). Overall, the torso showed a relatively higher RSR than the extremities at both intensities, and the RSR decreased from the medial to lateral across the torso. The increase in exercise intensity resulted in the increase in RSR in nearly all regions, except for feet and ankles, but has little impact on the whole-body sweat distribution.^41^

Adopting a similar measurement approach, the sweat distribution across the whole body, with the exception of the head, was studied targeting differences between young Caucasian females and males,^40^ and between Western European young and older males.^43^ In the comparison between genders, participants were requested to run on a treadmill at two exercise intensities (VO_2 max_ at 60% and VO_2 max_ at 75%). It was found that for female participants, the medial upper back (7) shows the highest RSR at the lower intensity, whereas the bra triangle (b) tops at the higher intensity. For male participants, the top three showing the highest RSR are medial mid back (9), medial upper back (7), and lower back (12) at both intensities, consistent with the previous results targeting solely young males.^40^^,^^41^ The comparison showed similar sweat distribution in the groups of different genders, with relatively higher RSR at the medial back in the torso and lower RSR toward the extremities. Two exclusive features were found in the female group: the first is the significant role of the bra triangle area in RSR at both intensities, and the second is the addition of heels and dorsal foot to relatively higher RSR regions at the lower exercise intensity.^40^ Likewise, the sweat distributions on the whole body (except for the head) of young and older males at rest and during physical exercise were investigated.^43^ The exercise intensity was controlled at a metabolic heat production of 200 W/m^2^. The comparison showed that RSR in the older is significantly lower than the younger group in almost all body regions at rest and specific regions (hands, legs, ankles, and feet) during exercise. In both groups during exercise, the highest RSR was found at the posterior torso, the upper and medial regions (7, 8, 9, 12) in particular, and the lowest RSR was observed at the feet region.^43^ In both studies, the head regions were excluded, and the massive sweating at the areas below the anterior and posterior neck and at the axilla (marked in gray in the figure) was collected but discarded.

Other studies have focused on the upper body sweat distribution when subjecting participants to exercise-related hyperthermia.^27^^,^^36^^,^^42^ Despite differences in forms of motion and participant selection, similar conclusions were made: (1) Among torso regions, the medial back along the spine shows the highest RSR, with the lowest toward the periphery, and a medial to lateral decrease across both the anterior and posterior torso; (2) The RSR in the posterior torso is overall higher than the anterior torso; (3) the upper-body sweat distributions in males and females are alike, the major difference being a relatively higher RSR in the upper chest and lateral upper back of females. The reason may be attributed to the additional coverage of sports bras on the upper torso of females.^36^

It should be noted that discrepancies occur between studies because of differences in environmental conditions, participant selections, heat-stimulating protocols, exercise intensities, measurement approaches, and measurement sites. For example, to build an environment that stimulates passive sweating, measures were taken by rising ambient temperature, immersing participants’ lower leg and/or feet in a hot water bath, and clothing the participants with hot water-perfusion suits.^47^ To study exercise-induced sweating, participants are often asked to run on an inclined treadmill or cycling on a friction-braked ergometer in a chamber with controlled temperature and relative humidity.^27^^,^^36^ The combined influences of the thermal and exercise loadings on RSR have also been studied.^33^ All the different conditions and participants make it difficult to directly compare the sweating rates between studies.

### Advanced sportswear for sweat management

Sportswear, worn next to skin, is in direct contact with human perspirations during heat-stimulated sports activities. Traditional hydrophilic fabrics such as cotton are easily wetted by perspirations but hard to be dried, leading to the accumulation of moisture in the space between the clothing and the skin.^48^ Textiles composed of hydrophobic synthetic fibers such as polyester, on the other hand, possess limited moisture absorption and water transportation capability, thus easily generating a hot and sticky feeling during active sports activities.^49^ To address these problems, profiled fibers such as Coolmax have been developed for use in sportswear featuring the quick-dry function. They possess superior moisture-wicking properties because of their increased surface area and the resultant capillary action.^50^^,^^51^ However, their moisture wicking is bidirectional, which means that water can be transported from the environment to the skin and vice versa. This limits their sweat-managing function in a highly humid climate or when caught by rain.^52^ In this context, directional water transport has become the key to resolving the issue of excessive sweat accumulation in sportswear.

The first theory to obtain directional fluid transport is to create asymmetric wettability between the two faces of fabrics. One way is through the selection of yarns and the design of fabric structures. Usually, hydrophobic yarns and hydrophilic yarns are used for the inner side and the outer side respectively to create the directional water penetration in fabrics. Differently, a bilayer fabric was proposed with hydrophobic polyethylene terephthalate (PET) yarns inside and moisture-responsive yarns outside.^53^ The moisture-responsive yarn consists of multiple bimorph fibers composed of hydrophobic triacetate and hydrophilic cellulose. Under dry conditions, the fiber components are loosely packed but become dense after sweat absorption, leading to an enlarged loop size in the knitted fabric and thus better ventilation.^53^ Lately, the effects of yarn type and knit structure in the double-face knitted fabrics on water absorption, moisture transfer, and liquid diffusion have been studied,^54^^,^^55^^,^^56^ revealing more possibilities in tuning moisture management via knit designs.

The other approach is through chemical treatment. A simple way to achieve asymmetric wettability is to apply a hydrophobic finishing on one face of a hydrophilic fabric or a hydrophilic finishing on one side of a hydrophobic fabric.^57^^,^^58^ An alternative to the whole single-face treatment was to create wicking channels by printing hydrophobic patterns on the in-contact-with-skin side of the cotton fabric,^59^ or to apply successive ultra violet (UV) irradiation to the fabrics with superhydrophobic coating (containing TiO_2_ and hybrid silica).^60^

**Figure 3:**
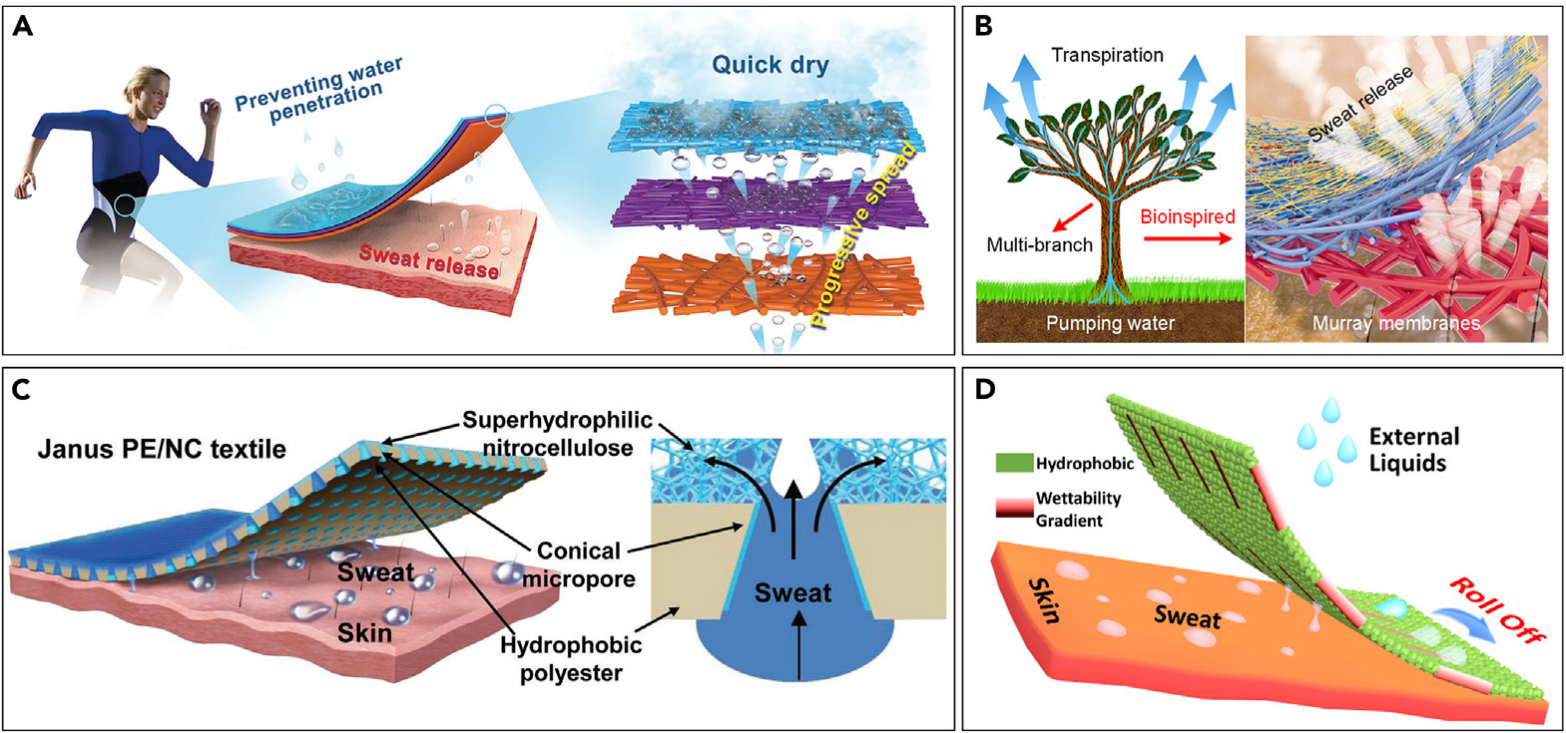
Advanced sweat moisture transport techniques (A) Tri-layered hydrophobic/transfer/super-hydrophilic fibrous membrane. Reproduced with permission from (Miao et al., 2018) Copyright© 2018, John Wiley and Sons. (B) Hydrophobic/super-hydrophilic Janus textile with asymmetric hydrophilic conical micropores. Reproduced with permission from (Dai et al., 2019) Copyright© 2019, John Wiley and Sons. (C) Layered fibrous membrane with hierarchical structure. Reproduced with permission from (Wang et al., 2018) Copyright© 2018, American Chemical Society. (D) Hydrophobic fabric with gradient wettability in discrete localized areas. Reproduced with permission from (Zou et al., 2021) Copyright© 2021, Elsevier.

Taking inspiration from the water-collecting and droplet-transporting abilities in natural creatures, such as desert beetles,^61^ cactus spine,^62^ spider silk,^63^ shorebirds’ beaks,^64^ and butterfly wings,^65^ more strategies have been developed to introduce the directional water transport function into textiles. In 2018, Miao et al. reported a tri-layered hydrophobic/transfer/super-hydrophilic fibrous membrane that can spontaneously move the sweat moisture away from the skin while blocking water penetration from outside to inside, as shown in Figure 3A^66^ The tri-layered membrane was produced using the electrospinning technique, composing a PU membrane, a hydrolyzed polyacrylonitrile-Si02 (HPAN) membrane, and a PU-HPAN membrane as the inner layer, outer layer, and transfer layer respectively. In this way, a progressive wettability featuring the combination of Janus wettability and wettability gradient was induced into functional moisture-wicking textiles.^66^ A novel type of hydrophobic/super-hydrophilic Janus textile with asymmetric hydrophilic conical micropores was fabricated by Dai et al., as shown in Figure 3B.^48^ A commercial Janus hydrophobic polyester (PE)/super-hydrophilic nitrocellulose (NC) membrane was used directly for laser perforation, to form a regularly hydrophilic conical micropore array. It showed a dynamic water transport from the PE layer to the NC layer and a reversal blockage.^48^ Inspired by the antigravity water transport and transpiration in vascular plants, as shown in Figures 3A and 3C synergistic action of a multi-branching porous structure and surface energy gradient was utilized to develop the quick-dry textiles.^52^ Following Murray’s law, the hierarchical structure was fabricated by layer-by-layer deposition of fibrous membranes with macro-, micron-, and submicron-sized pores.^52^ The addition of single-side electrospray of fluorinated PU with low surface energy enabled the introduction of a surface energy gradient in the fibrous Murray membranes.^52^ More recently, a fabric mimicking the back skin of desert beetles was developed by inducing gradient wettability in discrete localized areas to a hydrophobic fabric via plasma etching, as shown in Figure 3D.^67^ Sweat moisture from the skin can be transported to the outer surface through the localized areas with gradient wettability and accumulated to be larger droplets that can roll off the fabric.^67^ The limitation of the directional sweat transport by way of the gradient or asymmetric wettability is that the hydrophilic layer can no longer absorb water on saturation, which means that this system may not function well when exposed to prolonged and/or profuse sweating. This intrigues the development of a Janus textile-based electroosmotic pump with active directional water transport.^68^ Electroosmosis is a phenomenon of liquid flow when an electric field is applied across a porous dielectric material.^69^ Based on the electroosmotic pump mechanism, Zhang et al. developed a three-layer laminated material composed of a carbon-fiber woven fabric as the anode layer, an intermediate track-etch polycarbonate membrane, and a nickel-plated nonwoven fabric as the cathode layer, which were bonded together using thermoplastic polyurethane hot-melt mesh film.^68^ The application of an electrical field between the two electrode layers causes the sweat to move from the carbon-fiber fabric (positive electrode) to the nickel-plated nonwoven fabric (negative electrode). Their device showed a high flow rate (up to 20.81 mg min^−1^·cm^−2^·V^−1^) at low voltage (1–6 V), and the combination with insoles and sportswear illustrated the application possibility in real-life situations.

Novel textile or fibrous materials with sweat-managing ability are under continuing development. The theories behind sweat management in textiles are focused on the unidirectional water transport via Janus wettability, wettability gradient, or a combination of both. Further exploration by exploiting the electroosmotic pump mechanism provides new ideas on active sweat transportation in sportswear. The main purpose is to provide excellent physical comfort for the wearer under the circumstance of substantial sweating during active activities. Nevertheless, wearer comfort and product sustainability are often ignored in these studies, and the added functionality tends to make the products less recyclable because of the use of fiber blends and surface chemical treatments.

## Odor control in sportswear

### Evaluation techniques

To demonstrate the effects of textile materials on the odor-control function in sportswear, various odor evaluation techniques have been used by researchers, which can be broadly classified into the sensory method and the instrumental method. The sensorial technique involves a panel of assessors, trained or untrained, to determine the odor concentration, odor intensity, and hedonic tone.^70^ A standardized way of odor measurement using the human nose as the detector is called dynamic olfactometry.^71^^,^^72^ It involves first collecting odorous samples in suitable bags and then diluting them with odorless air at precise ratios on an instrument named an olfactometer. The analysis is conducted by presenting the diluted air to the panel, members of which are pre-screened with reference gases, at increasing concentrations of the odorous sample until the assessors can detect an odor. The dilution factor when reaching the odor threshold represents the odor concentration of the sample and is expressed as ou_E_/m^3^ according to EN 13725. It has been argued that the laboratory setting of dynamic olfactometry makes it unreliable to predict people’s real-world reaction to odor, because various perceptual and physiological responses may occur when the human body is entirely exposed to odor in real life.^73^ But the highly controlled condition of this method is considered scientific by many and has been put in use for odor assessment in industries like food, beverages, and perfumes.^74^^,^^75^^,^^76^ In a less standardized sensory trial, a panel of consumer assessors, none of whom is either trained or screened before assessing, can also be selected for odor evaluation. In this case, it is closer to the real-life setting and can reveal how ordinary consumers react to odors.^77^ Subjective ratings of odor intensity on a scale from not perceptible to extremely strong and hedonic tone on a scale from extremely unpleasant to extremely pleasant were also used as indexes. With this approach, the odor is perceived as a whole package instead of one or several chemicals, and can straightforwardly reflect how humans feel about it. But its deficiency is obvious, in terms of subjectivity, individual variability, fatigue, less sensitivity under prolonged exposure, and the mental state of panel members.^78^^,^^79^

The instrumental methods, on the other hand, appear more objective, standardized, and scientific, and are usually used to separate, quantify and/or identify the chemical compositions. A common procedure for odor analysis involves three steps: sample preparation and injection, separation, and detection. Diverse combinations have been used for VOC measurements regarding odor formation in textiles, such as GC-MS,^80^ GC-FID,^81^ SPME-GC-MS,^82^ and SPME-GC×GC-TOFMS.^83^^,^^84^ The most widely used combination is GC-MS because of its high sensitivity and reliability. But its most notable problem is its temporal resolution. The preconcentration process of the analyte sample and the separation of constituents through a capillary column are time-consuming. To increase the measuring speed for gaseous VOCs, proton-transfer-reaction mass spectrometry (PTR-MS) was developed as an alternative.^85^ It allows for online measurements of trace components and doesn’t require pre-treatment of the sample. But it only determines the mass of product ions, and doesn’t enable absolute identification of VOCs.^86^ This technique has been used for odor evaluation of textiles under the condition that the source of VOCs in the gaseous mixture is available from the literature or is already known.^87^^,^^88^^,^^89^^,^^90^ Other techniques that have been reported include liquid scintillation counting and infrared (IR)-based spectrometer. The former was used to measure the adhesion/release of ^14^C-labeled isovaleric acid, a representative substance of sweat odor, to/from knitted fabrics^91^; and the latter was utilized to quantify the adsorption/emission of four representative VOCs (acetone, ammonia, acetic acid, and butyric acid) in polyester/wool blend fabrics.^92^ The sensory technique can be used solely to compare the odor intensity of various textile materials or be combined with instrumental techniques to provide a more quantified and comprehensive analysis (Table 1).

**Table 1:** Different odor evaluation approaches targeting textile materials

Source	Evaluation method	Test conditions	Sample storage conditions	Textile materials compared
McQueen et al.^93^	Sensory	Wear trial	At controlled R.H. & Temp., 1/7/28 days (40 ± 5% R.H./65 ± 5% R.H., 20 ± 2°C)	WO, CO, PES
McQueen et al.^87^	PTR-MS	Wear trial	At controlled R.H. & Temp., 1/7 days (65 ± 5% R.H., 20 ± 2°C)	WO, CO, PES
Hammer et al.^91^	Liquid scintillation counting; Sensory	Isovaleric acid as representative VOC	–	WO, CO, PES
Prada et al.^82^	SPME-GC-MS	12 representative VOCs	–	WO, CO, PES, CV
Callewaert et al.^94^	Sensory	Wear trial	At room Temp. (20°C) in the dark, 28 h	CO, PES, CO/Synthetic
Rathinamoorthy et al.^95^	Sensory	Wear trial	Non-specified	CO, CV, LI, PA, 60/40 CO/PES, PES
McQueen et al.^83^	Sensory; SPME-GC×GC- TOFMS	Wear trial	Controlled R.H. & Temp., 12-14 h (65 ± 5% R.H., 20 ± 2°C)	CO, PES
Yao et al.^88^	PTR-MS	6 representative VOCs	–	WO, CO, PES
Rathinamoorthy et al.^80^	GC-MS	Wear trial	Non-specified	CO (herbal-extract-treated vs. untreated)
Klepp et al.^77^	Sensory	Wear trial	Non-specified	WO, CO, PES
de la Mata et al.^84^	SPME-GC×GC-TOFMS	Wear trial	Controlled Temp. (4°C) in the dark, R.H. & time not specified	CO, PES
Rathinamoorthy et al.^96^	Sensory	Wear trial	Non-specified	PES (alkali-treated vs. untreated)
Abdul-Bari et al.^81^	Sensory; GC-FID	Wear trial; 2 representative VOCs	(1) In a freezer at −20°C; (2) At room Temp. (65% R.H., 20°C), 7 days	PA, PES
Richter et al.^89^	PTR-MS	6 representative VOCs	–	WO, CO, PES
Wang et al.^92^	IR-based spectrometer; Sensory	4 representative VOCs;Wear trial	At room Temp. in the dark, 24 h	Varying PES/WO ratios
Richter et al.^90^	PTR-MS	11 representative VOCs	–	CV, WO, CO, PES

p.s. WO-wool; CO-cotton; PES-polyester; CV-Viscose; LI-linen; PA-nylon; CO/Synthetic-mixed cotton/synthetic; CO/PES-mixed cotton/polyester; PES/WO-mixed polyester/wool.

### Role of textile materials

It has been mentioned in previous sectors that body odor arises from the synergy of secretions from the human skin, and clothing, especially sportswear, would easily generate an unfavorable odor when soaked with sweat. An experimental study as early as 1953 has identified the contributing role of clothing in the production of axillary odor.^9^ Odor intensity and type may differ between the fabric substrate and the human body, and the clothing potentially contributes more to odor intensity.^93^ Considerable effort has been devoted to revealing the differences in odor profile between the fabric substrate and the human body and between different textile materials.

**Figure 4:**
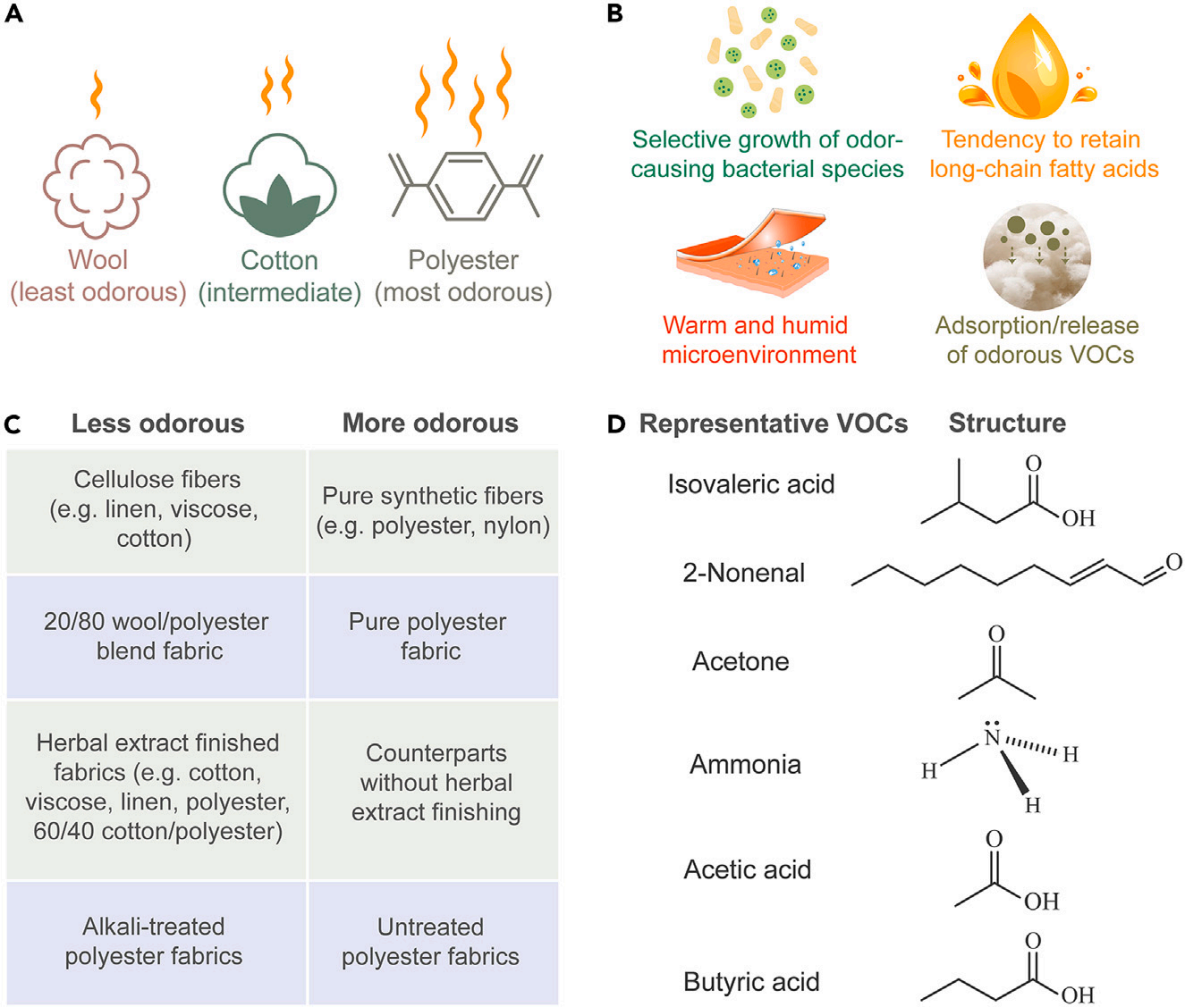
Role of textiles in odor generation (A) Comparison of wool, cotton, and polyester fibers in odor generation. (B) Possible explanations for why polyester smells more than wool after sweating. (C) Comparison of a diverse variety of fiber materials in odor retention. (D) Six representative VOCs used for evaluating the adsorption/desorption of malodor on various textile materials.

A study in 2007 investigated the effects of fiber type (cotton, wool, polyester) and knit structure (1 × 1 rib, interlock, single jersey) on the axillary odor intensity of apparel fabrics, and further the link with bacterial populations.^93^ Through sensory analysis on fabrics after a wear trial, the influence of fiber type was found to be the strongest, with polyester having the highest rating in odor intensity, wool the lowest, and cotton in the low level, slightly higher than wool fabrics (Figure 4A).^93^ Polyester fabrics with singer jersey structure showed a less intense odor than those with interlock and 1 × 1 rib structures, which was attributed to its thinner and lighter characteristic and the resultant better permeability. However, the difference in wool and cotton fabrics with different knit structures was imperceptible because of their intrinsically low-intensity rating.^93^ In 2014, a detailed investigation was done on the microbial growth and odor development in cotton and synthetic (mainly polyester) fabrics after a wear trial.^94^ Consistent with the previous conclusions, a significantly more intense and less pleasant smell was determined in polyester fabrics than in cotton fabrics by a trained panel. What is more, large differences were found in the microbiome profile between the axillary skin and textiles, and also between the cotton and synthetic clothing. As for the microbiome on axillary skin versus on textiles, what they have in common was *Staphylococcus* spp.; differently, the other dominating microbiome on the skin was *Corynebacterium* spp., which was generally regarded as the most important species causing axillary odor, whereas the other prominent microbiome on textiles was *Micrococcus* spp., which was held responsible next to *Corynebacterium* species.^94^ It was also found that *Staphylococcus* spp. were detected in relatively large amounts in almost all fabrics, while *Micrococcus* spp. were predominantly identified in 100% polyester and mixed synthetic fabrics.^94^ It indicates that the selective growth of different bacterial species may account for the detected different odor intensities in cotton and polyester fabrics.

Two theories have been proposed to explain why polyester tends to be more odorous than cotton and wool (Figure 4B)^87^: (1) There exist no hydrogen bonding groups in the polyester polymer molecule, leading to its high hydrophobicity and lipophilicity. Long-chain fatty acids, abundant in the apocrine and sebaceous secretions, are thus easily attracted to polyester polymers and readily work as the precursor for microbial degradation. (2) Wearing hydrophobic polyester fabrics usually induces a warmer and more humid microenvironment, which is more favorable for bacteria metabolic activity. To elucidate the better odor control ability of wool fiber, we should look into its molecular structure. The hydroxyl groups and amino acid groups in wool fibers could offer sites for the adsorption of ammonia and acidic molecules, which may contribute to the better odor-controlling ability of wool.^87^^,^^92^ Also, an increase in the adsorption rate of acetic acid into the wool fibers was found with the increase of relative humidity (RH) from 20% to 80%, which was seemingly explained by a lowered glass transition temperature (*T*_*g*_) of wool under the action of moisture.^97^

A large quantity of chemical compositions has been identified in the complex mixture of VOCs from the human body and textile samples, varying between genders, ages, body sites, fiber types, and even the methods used to collect scent.^98^^,^^99^ Compounds found include alcohols, carboxylic acids, aldehydes, ketones, esters, aliphatic, aromatics, and thiol.^100^ By selecting one or several of the reported VOCs to represent the human odor (Figure 4D), quantitative investigations on the adsorption/desorption of malodor on various textile materials have been conducted. The isovaleric acid was selected as the representative substance in sweat odor to study its retention on polyester, cotton, and wool fabrics.^91^ Results showed the highest release in polyester after 3 h and 20 h, and improved odor retention by adding *β*-cyclodextrin finish to cotton and polyester fabrics.^91^ Wang et al. selected four substances (namely acetone, ammonia, acetic acid, and butyric acid) as representatives to investigate the odor adsorption and emission ability of wool/polyester fabrics with different blend ratios. It was found that the ammonia/acetic acid/butyric acid adsorption increased with the increase of wool ratio in the blend, and ammonia/acetic acid release decreased to equilibrium as wool percentage increased to 20%.^92^ The conclusion was drawn that the addition of wool at a ratio of 20% to a polyester fabric is sufficient enough to reduce body odor in sportswear. In another series of studies, the body odor matrix was developed by mixing multiple selected VOCs at an optimized ratio to ensure an adequate detection signal for each.^88^^,^^89^^,^^90^ The results confirmed the low release of VOCs from cotton and wool, but higher release from polyester, consistent with the earlier findings.^90^ The adsorption characteristics of wool, cotton, and polyester fibers varied given the different VOCs selected for analysis in this series of research.

To date, most research focused on the effects of various fiber types and contents (mainly cotton, wool, and polyester), some on textile finishing, but few on fabric structures. Possible explanations for varied odor-control properties of different textile materials include but are not limited to the reaction between functional groups in fibers and odorant compounds, selective growth of the bacterial species that contribute majorly to malodor, and the microclimate change between the clothing and human body.

### Management strategies

**Figure 5:**
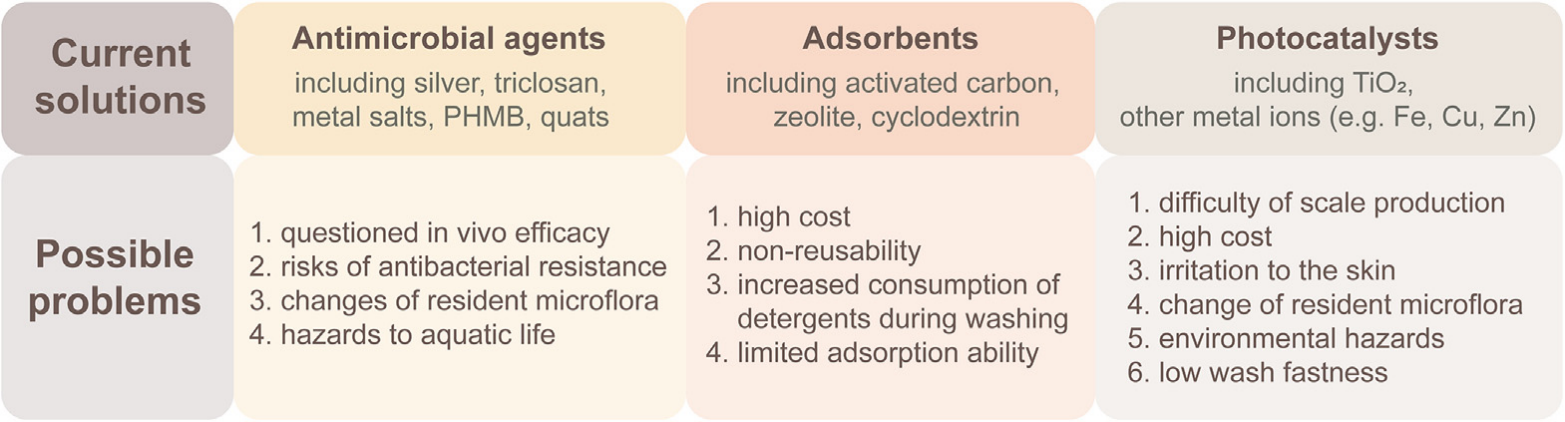
Current solutions and possible problems

There are three mainstream routes to resolve the odor issue in textiles: (1) To avoid the generation of odor from the source by eliminating the bacteria; (2) to adsorb the odorous chemicals using adsorbent materials; (3) to degrade the odorous compounds by way of photocatalysts. But each has its possible problems, as shown in Figure 5.

#### Antimicrobial-treated textiles

Given the crucial role of microorganisms in turning sweat from the initial odorless and sterile state to an acrid odorous state, imparting antimicrobial properties to fibers and/or fabrics has become the most common strategy to tackle odor issues. Antimicrobial agents used in commercial textiles include silver, triclosan, metal salts, polyhexamethylene biguanide (PHMB), and quaternary ammonium compounds (quats). Methods to incorporate these antimicrobials into textiles vary depending on the agent and fiber type. There has been a mature market featuring antimicrobial fibers or textiles. However, the effectiveness of antimicrobial-treated fabrics in odor management might be limited,^77^^,^^101^ and the risk of antibiotic/antibacterial resistance remains controversial. Concerns that the abuse of antibiotics/antibacterials may lead to increased antibiotics/antibacterials-resistant mutants have been reported, and the rational use of drugs that affect the microbial world has been called on.^102^ Compared to the less worrying influence that the antimicrobial chemicals would induce changes in chemical and physical properties, such as strength, handle, and appearance, of the textile products, that the resident flora of the wearer could be killed by the antimicrobial compounds raises more concerns. Most importantly, the antimicrobial chemicals on textiles are likely to be released to nature by washing, which may harm aquatic life.

#### Adsorbent textiles

A different approach involves applying adsorbent finishes on textile materials to adsorb the odorous molecules. The odor-control behaviors of different fiber materials basically are all related to adsorption. The diverse functional groups on the surface of fiber materials and the functional groups of the odor molecules determine the adsorption behavior. In addition to the inherent adsorption property of fibers/fabrics, other adsorbers have been developed to endow textile materials an enhanced odor removal ability, such as activated carbon, zeolites, and cyclodextrins.^103^^,^^104^ Activated carbon is a highly porous substance and its most common form for commercialization is in powder and granules, which can be applied to fabrics through printing, coating, and impregnation.^104^ The other form of activated carbon fibers were developed featuring exceptional adsorptive capacity because of high specific surface area and high micropore volume.^105^ Advantages include (1) more rapid dynamic adsorption and desorption with less material, (2) more diversity in fabric structure, (3) a wide range of precursor fiber polymers. However, the drawbacks, such as difficulty to color, low wear comfort, and high cost, limit their application in daily apparel. Zeolites, a type of microporous crystalline aluminosilicate material, possess unique adsorption properties of gases and vapors because of their large external surfaces, but the odor control properties of textiles incorporating zeolites are little investigated. Cyclodextrins are a type of sustainable compound produced by the enzymatic degradation of sugar and starch, among which β-cyclodextrin and its derivatives are most used because they are cheap, easy to produce, less prone to cause sensitivity or irritation to the skin, and don’t have mutagenic effects. The techniques used to incorporate β-cyclodextrins into fabrics include dyeing, printing, padding, spraying, coating, impregnation, and grafting.^106^ The adsorption mechanism of cyclodextrin to VOCs can be described using host-guest inclusion complexes. Owing to the inherent hydrophobic cavity, cyclodextrin rings can trap the VOC molecules under non-covalent interactions such as van der Waals forces, H-bonds, and hydrophobic attractions.^107^ The eco-friendly nature of cyclodextrins makes them attractive substances for the textile industry, but their potential in endowing everyday wear with better odor-managing capabilities is yet to be exploited. Overall, research on tackling the sweat odor problem using adsorbers is rather little. Possible drawbacks include high costs, non-reusability of the adsorptive substances, increased consumption of chemical detergents during washing, and the release of adsorbed malodor after saturation or under elevated temperature.

#### Photocatalyst-containing textiles

Another alternative option is to convert odorous VOCs into odorless constituents by the use of photocatalysts. This technique has been considered promising in indoor air purification, and its application in the textile field has also been reviewed.^108^^,^^109^ Titanium dioxide (TiO_2_) is the most commonly used photocatalyst for either purpose. Substrate textile materials range from commonly used cotton and polyester fibers to glass fibers and acrylic/cellulose diacetate blend fibers.^110^^,^^111^^,^^112^^,^^113^ Target air pollutants include acetone, ammonium, benzene, ethylbenzene, formaldehyde, toluene, m-xylene, n-butyl acetate, etc.^109^ There are primarily two approaches to applying TiO_2_ on textiles, the first of which is to add TiO_2_ to the melt polymer of synthetic fibers before extrusion, and the other is to directly finish fabrics with TiO_2_ using padding, coating, or impregnation methods.^109^ Other metal ions such as Fe, Zn, and Cu have also been deposited on textiles for deodorizing investigations.^103^ But the photocatalytic oxidation reaction process can be influenced by many factors, such as ultraviolet source and intensity, pollutant concentration, humidity, and temperature.^108^ The possibility of using photocatalysts to reduce sweat odor in sportswear has not yet been exploited. Possible limitations include (1) increased cost because of the addition of metal ions, (2) challenges to realize scale production, (3) insufficient wash fastness, (4) impaired handle and appearance of fabrics, (5) irritative effects on human skin, (6) resultant changes of resident flora on skin, (7) environmental hazards.

In summary, there are plenty of drawbacks in current deodorant products, which call for the development of new odor-control textiles. The antimicrobial technique appears to be the most popular way, but concerns, including the risk of antibiotic/antibacterial resistance, effect on the resident flora of the human body, and the adverse impact on the environment, arise, not to mention the questionable effectiveness, and the likely impaired properties of textiles. Little research has been conducted on the influences of the adsorbent materials on the odor-managing property of textiles after exposure to perspiration. And the possibility of applying photocatalytic decomposition to resolve sweat odor issues in sportswear remains a puzzle.

### Conclusion and outlook

To sum up, body odor arising after sweating is a synergistic effect of skin secretions and bacterial degradation. The degradation of precursor compounds in perspiration by bacteria in the humid micro-environment leads to the production of odorous chemicals, the volatilization of which triggers the human olfactory sensing of malodor. The transferring and clinging of on-skin substances to sportswear make clothing a second source of the odor. Considering the significant role of sweat in odor formation, the sweat distribution of the human body and the advanced sweat-managing techniques were reviewed. Despite differences in sweating rate between genders, the sweat distributions of both are alike. To realize directional fluid transport in textiles, current methods include creating asymmetric wettability in Janus fabrics with hydrophobic inside and hydrophilic outside, inducing a wettability gradient through the thickness of fabrics, and forming Murray hierarchical structures. None of the sweat management developments in textiles, however, has addressed their effects on sweat-induced odor. Moreover, the odor in fabrics is usually assessed with the human sensory method, and/or instrumental method. Consensus is reached through experiments that wool shows the best odor adsorption and retention, closely followed by cotton, and polyester has been repeatedly shown to be among the worst odorous textile material. Other proposed influence factors embody textile finishing agents, the physical structure of fibers and fabrics, and microclimate change.

Based on different mechanisms, the solutions to malodor in sportswear are classified as antimicrobial, adsorbent, and photocatalyst degradation types. Similar to antimicrobial deodorants, antimicrobial-treated fabrics may impose hazardous impacts not only on aquatic creatures but also on the safety of humans themselves. The other two approaches, by contrast, are a long way from application in sportswear because of a lack of relevant research and possible drawbacks, such as high cost, impairment to comfort and physical properties, irritation to the skin, and safety issues especially when metal ions are used. In light of the progress made on sweat management and odor control in textiles, more need to be done to develop next-generation sportswear that exhibits superb odor elimination or suppression properties. To this end, the following areas warrant further research.

(1) It remains unclear whether or not the fabrics having superior sweat transport abilities could demonstrate an improved odor-control function. Further experimental analysis is needed to reveal the association between sweat-managing and odor-control properties of fabrics.
(2) The study on the influence of fabric structure variations on the odor adsorption/desorption of fabrics is very limited. It would be both cost-efficient and eco-friendly if the optimized combination of knit design and fiber selection could be determined for odor reduction in textiles.
(3) More plant-based substances and fibers with antibacterial actions could be exploited. The efficacy of sustainable cyclodextrin treatment on various textiles to adsorb sweat-induced VOCs could also be further explored.
(4) Considering the selective growth of bacteria on different textile materials, targeted antimicrobial agents could be taken into account to replace the wide-spectrum ones, to reduce their side effects on profitable strains.
(5) New sportswear design may be explored to use different fiber materials or treatments in specific target areas which are prone to sweat concentration and malodor development. For example, the unidirectional moisture transport properties of sportswear fabrics could be improved by novel yarn materials, knit structure designs, and surface modification of fibers or fabrics.
